# Development and Validation of a Gas Chromatography‐Mass Spectrometry Method for the Determination of Fentanyl and Butyryl Fentanyl in Oral Fluid

**DOI:** 10.1002/ansa.202400038

**Published:** 2024-12-23

**Authors:** Nicola Camedda, Sara Dagoli, Luca Anzillotti, Rossana Cecchi

**Affiliations:** ^1^ Department of Medicine and Surgery Legal Medicine University of Parma Parma Italy; ^2^ Department of Biochemical, Metabolic and Neural Sciences, Institute of Legal Medicine University of Modena and Reggio Emilia Modena Italy

**Keywords:** butyryl fentanyl, fentanyl, gas chromatography‐mass spectrometry, oral fluid, synthetic opioids

## Abstract

Synthetic opioids are lab‐synthesized substances that target the brain's opioid receptors, offering analgesic and sedative effects. Amongst them, fentanyl is one of the most widely used to intervene in chronic pain in moderate to severe cancer situations. Butyryl fentanyl (BF) is a novel synthetic opioid whose use is growing. Its potency is seven times that of morphine and, unlike fentanyl, BF can only be obtained through illegal sources. Fentanyl and its analogues are related to harmful intoxications and an increase in opioid‐related mortality in many countries, such as in the United States and Europe in recent years. This work developed and validated an effective and sensitive method based on solid‐phase extraction followed by gas chromatography‐mass spectrometry (GC‐MS) for the determination of fentanyl and BF in oral fluid samples. To the best of our knowledge, it is the first successful attempt to quantify these analytes using GC‐MS with a limit of quantification (LOQ) of 1 ng/mL in OF. Intra‐day and inter‐day percentage coefficient of variation were found within 1%–3% and 1%–14%, respectively, while accuracy ranged from 92% to 102% at four concentration levels (lower LOQ [LLOQ], 3, 20, 40 ng/mL) in accordance with the established criteria. The absolute recovery values were in the range of 80.0%–100.0%. The method was linear for all analytes, with quadratic regression of calibration curves always higher than 0.99. The validated method demonstrated its great potential to detect and quantify fentanyl and its analogue in OF and it can be useful not only in forensic investigations of addiction histories but also in epidemiological studies on the spread of fentanyl and BF among workers and/or drivers.

## 1 Introduction

Synthetic opioids are lab‐synthesized substances that target the brain's opioid receptors, offering analgesic and sedative effects. Amongst them fentanyl is one of the most widely used to intervene in chronic pain in moderate to severe cancer situations [1, 2], acting selectively on the μ‐opioid receptor with minor activity at the Δ and κ receptors [3, 4, 5].

Despite its therapeutic use, recently there has been a surge in its illicit circulation, leading to a rising risk of life‐threatening poisonings [6, 7]. Initially utilized as a heroin substitute, fentanyl's history of illicit abuse has persisted, contributing to a global increase in opioid‐related deaths, particularly in the United States [8, 9, 10]. The emergence of various fentanyl analogues, both for medical and non‐pharmaceutical use, reflects the complex landscape of opioid abuse. Sold in various forms on the illicit market, fentanyl poses a significant public health concern.

The abuse of synthetic opioids has increased in several countries worldwide and throughout the present decade and this abuse has resulted in epidemic‐level harms in some countries. Deaths from opioid use are increasing [6], especially in the US, with a growing percentage due to synthetic opioids.

Several analogues of fentanyl, which vary in potency and pharmacokinetic properties, were designed and modelled on the synthesis of the latter. While some of them were registered for human use (alfentanil, remifentanil, sufentanil and lofentanil) and in veterinary medicine for wild animals (carfentanil and thiofentanil), others (e.g. acetylfentanyl, acryloyfentanyl, butyrylfentanyl, cyclopentylfentanyl, furanylfentanyl and octofentanil)—also known by the name of non‐pharmaceutical fentanyls (NPFs)—never developed into a medicinal product [11, 12, 13, 14, 15].

As far as the illicit market is concerned, fentanyl is generally sold as a powder to dissolve or inject, smoke or inhale, as nasal sprays, liquids or tablet forms [16]. Butyryl fentanyl (N‐(1‐phenethylpiperidin‐4‐yl)‐N‐phenylbu‐tyramide or butyrfentanyl or BF) is a designer fentanyl and it was first reported in Poland in the summer of 2013 [17]. BF is a novel synthetic opioid whose use is growing [18]. Its potency is seven times that of morphine [2] and, unlike fentanyl, BF can only be obtained through illegal sources [19].

Intravenous administration of BF is the most common route, and its pharmacological profile can be regarded as being like fentanyl, morphine, and other synthetic opioids [20].

The original Janssen route to fentanyl could be central to the synthesis process of BF as the only difference between this opioid and the parent fentanyl one can be localized in the nature of the amide portion of the molecule. Another option is that it is made following the reaction between 4‐ANPP, the last intermediate before fentanyl in the Siegfried and Valdez routes, with butanoyl chloride [21].

During the last two decades, forensic toxicologists developed a series of analytical methods for detecting synthetic opioids with the intention of counteracting the ongoing opioid overdose epidemic. Standard targeted analytical techniques such as liquid chromatography‐tandem mass spectrometry (LC‐MS/MS) [22, 23] and gas chromatography‐MS (GC‐MS) [24, 25] were often used for the detection of synthetic opioids [26, 27, 28, 29, 30, 31, 32, 33, 34, 35].

The field of substances of abuse is ever‐changing and the interest in using oral fluid (OF) for forensic and toxicological purposes has grown significantly in recent years as a consequence of the advantages of this matrix, and also due to the extraction and analytical procedures having improved [36, 37, 38]. OF has gradually become popular as an alternative biological specimen for the detection of drugs [3, 39, 40, 41]. The use of alternative matrices in toxicological analyses has begun to characterize clinical and forensic settings [42] and, within this panorama, OF as non‐invasive fluid has attracted attention in the field of drugs [43, 44, 45, 46].

OF is characterised by easy and non‐invasive specimen collection. Their free fraction form is the modality in which drugs are usually present since the bounded drug may not infiltrate through the salivary tissues [47].

Regarding the analysis of fentanyl and BF, several examples of GC‐MS analytical methods were reported in the literature and many improvements in detecting low concentrations of fentanyl derivatives have been carried out. However, most of them were related to urine and blood specimens. In 201,3 Strano Rossi et al. reported an analytical method for the quantitative detection of fentanyl and their metabolites in urine [25]. In 2019, Misailidi and co‐workers reported a method validation of synthetic opioids, including BF in blood [35]. Although the improvements related to quantifying these analytes at low concentrations in blood and urine employ the GC‐MS technique, OF remains an unexplored matrix. Moreover, BF is less studied compared to other synthetic opioids and there are no cases in which BF was detected employing GC‐MS in OF. Fentanyl is generally more investigated in urine and blood, but only one case has been reported for its detection in OF implementing GC‐MS as an analytical instrument [33]. This led us to develop an analytical method for the detection and quantification of fentanyl and BF in OF employing a GC‐MS technique.

This method requires the use of solid‐phase extraction (SPE) as a useful tool for a straightforward pre‐treatment of samples employing fentanyl *d_5_* as an internal standard. Based on the limited published scientific literature, currently, there are no examples of validated analytical GC‐MS methods detecting fentanyl and BF at very low concentrations in OF.

## 2 Materials and Methods

### 2.1 Chemicals and Materials

Reference standard solutions in methanol of fentanyl and BF were used to prepare the corresponding working solutions. BF 1 mg/mL solution was purchased from Cayman Chemical (Michigan, USA). A standard solution containing 1 mg/mL of fentanyl was acquired from LGC Standards (Milan, Italy). Fentanyl *d_5_* was obtained from Lipomed (Basilea, Switzerland) at a concentration of 1 mg/mL. Sodium acetate and methanol were acquired from Carlo Erba reagents (Milan, Italy). Twenty drug‐free OF samples were obtained from male and female volunteers and used for the preparation of calibration curves. SPE Strata X Drug B 33 mm Polymeric strong Cation cartridges were purchased from Phenomenex s.r.l (Bologna, Italy). Ethyl acetate was acquired from ITW Reagents division (Illinois, USA) while isopropanol and ammonia from Carlo Erba reagents (Milan, Italy).

### 2.2 Calibration and Sample Extraction

Working solutions containing both 0.7 µg/mL of fentanyl and BF, and 1 µg/mL of fentanyl *d_5_* were used for the preparation of the spiked OF samples at concentrations of 0.5, 1.0, 2.0, 5.0, 10, 20, 25, and 50 ng/mL. For QC samples a different working solution containing 0.7 µg/mL of fentanyl and BF was prepared. Standard solutions and spiked samples were stored at −20°C until use. Extraction of analytes was carried out with Strata X Drug B 33 mm Polymeric strong cation cartridges. Before loading to the cartridges, 10 µL of 1 µg/mL fentanyl *d_5_* working solution were added to 2 mL ± 0.5 OF and then samples were diluted with 2 mL of 0.1 M acetate buffer solution pH 5.

Saliva was collected by spitting in the absence of stimulation. Oral fluid samples were collected from 20 healthy volunteers free of drugs of abuse (both males and females), after obtaining their informed consent. During collection, salivary samples were transferred to a plastic tube with an identification number in order to avoid any possible identification of the donor. Each sample was centrifuged at 4000 rpm for 10 min. Five different samples randomly chosen between males and females were mixed in order to obtain a pooled OF sample. The pooled lots were aliquoted and stored at −20°C.

The samples were then loaded onto the SPE cartridges. The extraction was conducted at a speed of 1 drop/s. The column was washed with an additional 2 mL of 0.1 M of acetate buffer solution pH 5, methanol (2 mL) and dried under a stream of nitrogen for 10 min. Elution of analytes was performed with a 70:20:10 mixture of ethyl acetate: isopropanol: ammonia hydroxide (750 µL twice). The eluates were then dried under nitrogen at 40°C, reconstituted with 50 µL of ethyl acetate and injected into the instrument. Fentanyl and BF were analyzed in GC‐MS without derivatization.

### 2.3 GC‐MS Analysis

**FIGURE 1:**
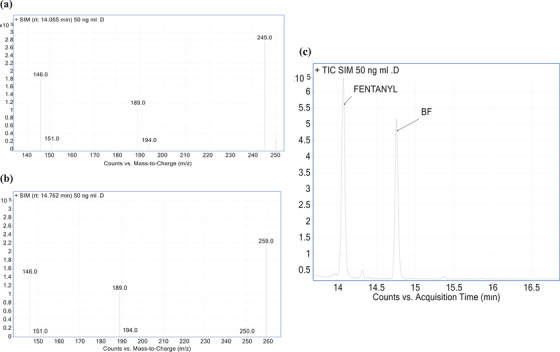
(a) Mass spectra in selective ion monitoring (SIM) mode of fentanyl (14.05 rt), (b) butyryl fentanyl (14.7 rt) at 50 ng/mL, and (c) fentanyl and butyryl fentanyl chromatogram at 50 ng/ml.

GC‐MS analyses were carried out on an Agilent 7820A gas chromatograph coupled to a 5977B single quadrupole mass spectrometer (Agilent Technologies, Waldbronn, Germany), operating both in selective ion monitoring (SIM) and in Scan modes (scan range 50–550 amu). Acquisition and data analysis were performed using standard software supplied by the manufacturer. The column was an HP‐5MS (5% Diphenyl/95% Dimethylpolysiloxane) capillary column (30 m × 0.25 mm. i.d., 0.25 µm film thickness, Agilent Technologies). The temperature program was as follows: 100°C, 42°C/min to 200°C, hold for 2.67 min, 15°C/min to 280°C, hold for 12 min. The injection port and ion source temperatures were set at 250 and 230°C, respectively. Split injection mode with a split ratio of 100:1 was used, and helium was employed as carrier gas at a flow rate of 0.7 mL/min. The mass spectrometer (MS) was operated in the electron ionization (EI) mode (70 eV). The mass spectra of the analytes were recorded by total‐ion monitoring to determine retention times (RTs) and characteristic mass fragments. For quantitative analysis, the chosen diagnostic mass fragments were monitored in SIM mode. The analytes were initially analyzed in scan mode (50–500 *m/z*) using EI. The spectrum of each analyte was compared with reference spectra available in the NIST MS program. One quantifier and two qualifier ions per fentanyl were used for their determination into the matrix (Table 1 and Figure 1).

**TABLE 1:** Retention times (in minutes) and diagnostic ions in *m/z*. Ions in bold are used for quantitation.

Compound	Retention time (min)	Quantifier (*m/z*)	Qualifier 1 (*m/z*)	Qualifier 2 (*m/z*)
Fentanyl	14.05	**245**	146	189
Butyryl fentanyl	14.7	**259**	146	189
Fentanyl *d_5_*	14.05	**250**	151	194

### 2.4 Method Validation

The analytical method was validated according to ICH guidelines [48] and it was evaluated for linearity, the limit of detection (LOD), the limit of quantification (LOQ), accuracy, precision, selectivity, specificity, carryover and recovery. Calibration curves were plotted in triplicate on three different days by adding to blank OF samples aliquots of a working solution containing fentanyl and BF at 0.7 µg/mL. Repeatability and accuracy were studied at four concentration levels: LOQ, low (QC1), medium (QC2) and high (QC3). All parameters studied are listed below.

### 2.5 Interferences

Twenty negative OF samples were collected from male and female subjects and analyzed for interfering peaks. OF samples were fortified with aliquots at the concentration of 50 ng/mL of common drug of abuse (cocaine and related metabolites coca‐ethylene and benzoylecgonine, common opiates such as morphine, methadone, codeine, dihydrocodeine and benzodiazepines such diazepam, clonazepam, flunitrazepam, lorazepam and nitrazepam) to evaluate selectivity. Satisfactory selectivity was established if no interfering signals were detected in terms of characteristic fragments at the RTs of analytes related to endogenous or exogenous compounds.

### 2.6 LOD and LOQ

The LOD was expressed as the concentration producing a signal‐to‐noise (S/N) >3 for at least three ion fragments for each analyte. The LOQ was considered the concentration giving at least an S/N>10 for three ion fragments and acceptable accuracy and precision (percentage coefficient of variation [%CV], %E < 20%).

### 2.7 Linearity

The linearity of the method was studied in the range from the LOQ of each substance to 50 ng/mL. Calibration curves were plotted in triplicates on three different days. The curves were constructed by the method of least‐squares with a weighting factor of 1/x and linearity was expressed as quadratic regression coefficient (R^2^).

### 2.8 Accuracy and Precision

The accuracies of the method were expressed as the percentages of the systematic error (E%) and precisions as CV%. Oral fluids were fortified at 1.0 ng/mL (LOQ), 3 ng/mL (three times LOQ, QC1), and 20.0 ng/mL (30%–50% of calibration range, QC2) and 40 ng/mL (at least 75% of upper LOQ, QC3). The bias and precision of this method were measured in five replicates over three different days. Precision and accuracy were calculated based on the quantifier ions. Intraday precision was evaluated by injecting each QC sample five times daily into the instrument. Interday precision was evaluated by analyzing all QCs on three different working days.

### 2.9 Memory Effect

Three spiked OF samples at of 50, 100 and 200 ng/mL with fentanyl and butyryl fentanyl were prepared. All samples were extracted as described above and injected into the instrument (*n* = 3), along with solvent blanks. After each run of a fortified sample, a blank was evaluated to verify the presence of carryover, which was not present up to 200 ng/mL.

### 2.10 Recovery

The recovery was evaluated by comparing extracted samples at three different concentrations (low 5 ng/mL and medium, 20 ng/mL and high 50 ng/mL) with corresponding samples spiked after extraction.

## 3 Application of the Method

**FIGURE 2:**
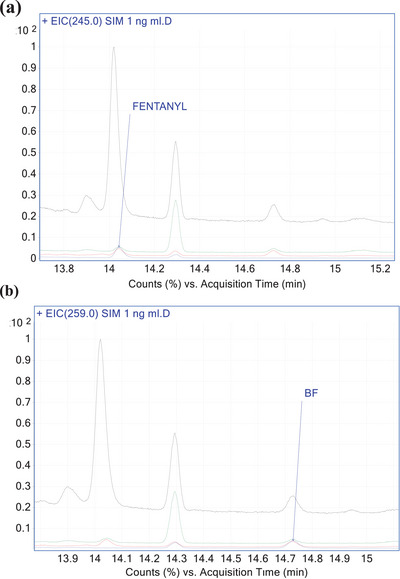
Chromatograms of an oral fluid sample spiked at 1 ng/mL fentanyl (**a**, 14.05 rt) and butyryl fentanyl (**b**, 14.7 rt) in selective ion monitoring (SIM) mode.

Thirty OF samples were analysed and collected from subjects aged 18–40 years old. The proposed method demonstrated its specificity for the detection of fentanyl and BF, verifying the absence of interfering signals at the RTs of the analytes (Figure 2a,b). Calibration curves were plotted for fentanyl and BF prepared from OF at concentrations of 0.5, 1, 2, 5, 10, 20, 25 and 50 ng/mL. The calculated calibration curves displayed excellent linearity (R^2^ ≥0.998) within the range of 1.0–50 ng/mL for both analytes. The LOQ was defined as the lowest concentration of the standard calibration with an S/N of at least 10 and acceptable criteria of inaccuracy and imprecision (± 20%). Hence, we set the LOQ at 1 ng/mL for both analytes and it was evaluated using five replicates per run, over 3 days with three different blank matrix sources. OF samples at concentrations of 0.3, 0.5 and 0.7 ng/mL were prepared for evaluating LODs in the chromatogram. All experiments were run in triplicate for each concentration. The LODs were 0.50 ng/mL for both analytes.

**FIGURE 3:**
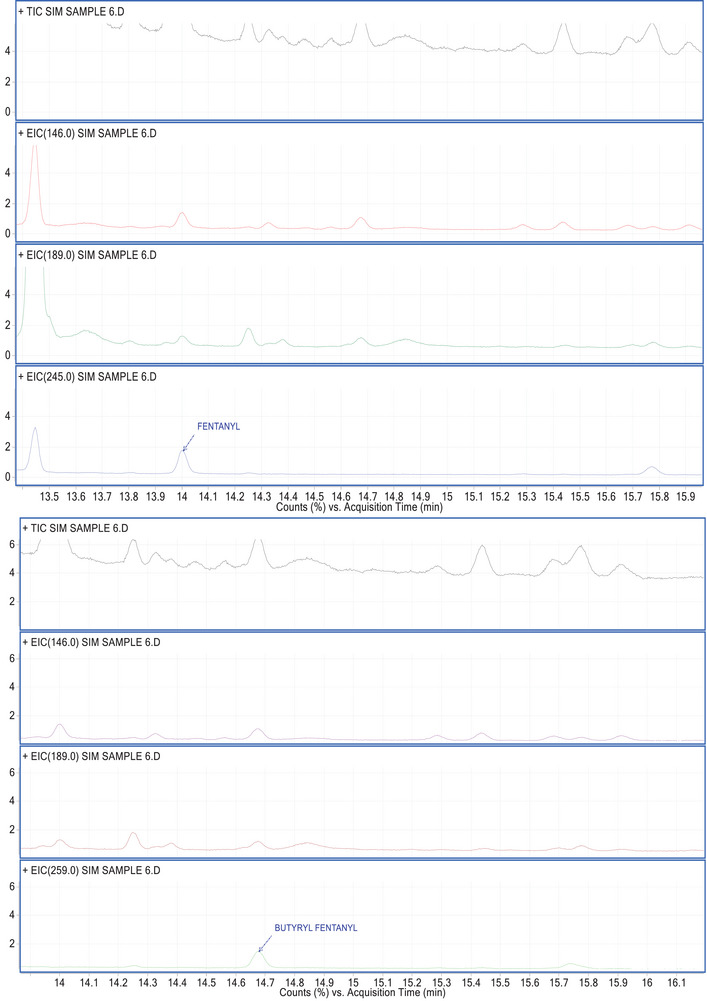
Chromatograms of a sample positive for fentanyl and butyryl fentanyl (BF).

The samples were collected and processed to maintain anonymity, and integrity and prevent contamination. The results of our investigation revealed varying levels of fentanyl and BF exposure among selected individuals, with five samples testing positive for fentanyl and/or BF. Concentrations quantified in these positive samples were 1.38, 1.55, 1,20, 1,07 and 2.02 ng/mL for fentanyl. Two of those samples displayed positivity also for BF, with concentrations of 1.65 and 1.93 ng/mL. A chromatogram of a positive sample of fentanyl and BF is reported in Figure 3. These findings provide critical data on the prevalence and potential risks associated with both analytes' use in this demographic.

## 4 Results and Discussion

Due to the increasing abuse worldwide of fentanyl derivatives, it is necessary to develop useful analytical methods for their detection in different biological matrices. This GC‐MS method was meticulously developed to reach high selectivity, which is crucial for accurately identifying and quantifying analytes at trace levels in complex biological matrices such as OF. Our validation process ensured the reliability and reproducibility of the method, conforming to stringent analytical standards.

The results are shown in Table 2. Accuracy and precision were defined, respectively, as CV% and E% and the results are reported in Table 3. Values are lower than 20% for low concentrations and lower than 15% for high concentrations. Accuracy and repeatability were excellent for all analytes at their respective LOQs. Hence, according to the guidelines, this method displayed acceptable accuracy and precision values. Recovery was high and varied from 80 to 100% as shown in Table 3. CV% related to intra‐day was between 0.8 and 2.5% for fentanyl and between 1.6 and 2.5% for BF. Between runs, precision varies from 6% to 12% for both analytes. In summary, validation parameters such as LODs, LOQs, precision, accuracy and linearity make this method adequate for the analysis of fentanyl and BF in OF.

**TABLE 2:** Validation data, limits of detection (LODs), limits of quantification (LOQs) and linearity.

Analyte	LOD (ng/mL)	LOQ (ng/mL)	Slope (±SD)	Intercept (±SD)	R^2^ (±SD)	Slope CV%	R^2^ CV%
Fentanyl	0.5	1	1.333 ± 0.0320	−0.0391± 0.020	0.999 ± 0.0004	2.4	0.04
Butyryl fentanyl	0.5	1	1.245 ± 0.037	−0.0372 ± 0.017	0.998 ± 0.0009	3.0	0.1

**TABLE 3:** Intra‐/inter‐day precision, percentage of systematical error and recovery at 5, 20 and 50 ng/mL for each analyte.

Analyte	QCs Concentration (ng/mL)	Intraday precision (CV%)	Interday precision (CV%)	Accuracy (E %)	Recovery at 5, 20 and 50 ng/mL (%)
Fentanyl	LLOQ 3 20 40	0.84 1.6 2.5 2.2	13.8 1.6 4.26 6.14	2.3 −0.04 −3.8 −7	89 100 80
Butyryl fentanyl	LLOQ 3 20 40	2.6 3.4 1.6 2.6	12.3 12.8 5.6 9.1	−0.08 −0.94 −7.24 −8.45	86 100 82

## 5 Conclusion

Blood and urine are generally the first choice of samples to be tested for toxicological assessments in different contexts. However, due to its ease of sampling and blood‐similar window of detection, OF could be a good alternative matrix for detecting synthetic opiates. This analytical method reported herein is straightforward, selective, and accurate for the determination of these analytes in OF and it can be useful not only in forensic investigations of addiction histories but also in epidemiological studies on the spread of fentanyl and BF among workers and/or drivers. Notably, it is proposed as the first attempt to quantify fentanyl and its derivative BF using GC‐MS with a LOQ of 1 ng/mL. Future perspective could be a further extension of this method to other synthetic opiates and its application to real cases.

The presence of fentanyl in these real samples underscores significant public health concerns, emphasizing the need for enhanced surveillance, early intervention, and targeted educational programs to address and mitigate the risks of fentanyl exposure among young individuals.

Our study contributes to the growing body of evidence necessary for informing policy decisions and developing effective preventive strategies to combat the opioid crisis.

## Author Contributions

**Camedda Nicola**: Conceptualization, data curation, methodology. **Dagoli Sara**: Conceptualization, data curation, formal analysis, methodology. **Luca Anzillotti**: Conceptualization, data curation, methodology. **Cecchi Rossana**: Conceptualization, data curation, formal analysis, methodology.

## Conflicts of Interest

The authors declare no conflicts of interest.

## Funding

This research did not receive any specific grant from funding agencies in the public, commercial, or not‐for‐profit sectors.

## Data Availability

The data that support the findings of this study are available from the corresponding author upon reasonable request.

## References

[1] M. P. Davis , “Fentanyl for Breakthrough Pain: A Systematic Review,” Expert Review of Neurotherapeutics 11 (2011): 1197–1216. 21797660 10.1586/ern.11.63

[2] M. J. P. Geist , V. C. Ziesenitz , H. J. Bardenheuer , J. Burhenne , G. Skopp , and G. Mikus , “Minor Contribution of Cytochrome P450 3A Activity on Fentanyl Exposure in Palliative Care Cancer Patients,” Scientific Reports 9 (2019): 14635. 31601999 10.1038/s41598-019-51279-6 PMC6786992

[3] S. R. Bista , A. Haywood , R. Norris , et al., “Saliva versus Plasma for Pharmacokinetic and Pharmacodynamic Studies of Fentanyl in Patients With Cancer,” Clinical Therapeutics 37 (2015): 2468–2475. 26404396 10.1016/j.clinthera.2015.09.002

[4] H. Pathan and J. Williams , “Basic Opioid Pharmacology: An Update,” Br J Pain 6 (2012): 11–16. 26516461 10.1177/2049463712438493 PMC4590096

[5] J. Shim , A. Coop , and A. D. MacKerell , “Molecular Details of the Activation of the M Opioid Receptor,” Journal of Physical Chemistry B 117 (2013): 7907–7917. 23758404 10.1021/jp404238n PMC3735350

[6] J. K. O'Donnell , J. Halpin , C. I. Mattson , B. A. Goldberger , and R. M. Gladden , “Deaths Involving Fentanyl, Fentanyl Analogs, and u‐47700‐10 States, July‐December 2016,” Morbidity and Mortality Weekly Report 66 (2017): 1197–1202. 29095804 10.15585/mmwr.mm6643e1 PMC5689219

[7] Drug Enforcement Agency (DEA) , Counterfeit prescription pills containing fentanyls: a global threat. 2016, https://www.dea.gov/.

[8] Annual Review of Pharmacology and Toxicology , The opioid epidemic: crisis and solutions. 2018. Accessed November 25, 2024. https://www.annualreviews.org/doi/10.1146/annurev‐pharmtox‐010617‐052534. 10.1146/annurev-pharmtox-010617-052534 28968188

[9] N. D. Volkov and C. Blanco , “The Changing Opioids Crisis: Development, Challenges and Opportunities,” Molecular Psychiatry 26 (2021): 218–233. 32020048 10.1038/s41380-020-0661-4 PMC7398847

[10] J. Brent and S. T. Weiss , “The Opioids Crisis‐not Just Opioids,” JAMA Network Open 5 (2022): e2215432. 35657631 10.1001/jamanetworkopen.2022.15432

[11] T. H. Stanley , “The Fentanyl Story,” Journal of Pain 15 (2014): 1215–1226. 25441689 10.1016/j.jpain.2014.08.010

[12] J. Suzuki and S. El‐Haddad , “A Review: Fentanyl and Non‐pharmaceutical Fentanyls,” Drug and Alcohol Dependence 17 (2017): 107–116. 10.1016/j.drugalcdep.2016.11.033 28068563

[13] R. B. Raffa , J. V. Pergolizzi Jr., J. A. Le Quang , et al., “The fentanyl family: A Distinguished Medical History Tainted by Abuse,” Journal of Clinical Pharmacology and Therapeutics 43 (2018): 154–158. 10.1111/jcpt.12640 28980330

[14] B. Zawilska , “An Expanding World of Novel Psychoactive Substances: Opioids, Front,” Psychiatry 8 (2017): 110. 10.3389/fpsyt.2017.00110 PMC5492455 28713291

[15] K. Kuczyńska , P. Grzonkowski , L. Kacprzak , and J. B. Zawilska , “Abuse of Fentanyl: An Emerging Problem to Face,” Forensic Science International 289 (2018): 207–214. 29902699 10.1016/j.forsciint.2018.05.042

[16] D. Abdulahim and O. Bowden‐Jones , The Misuse of Synthetic Opioids: Harms and Clinical Management of Fentanyl, Fentanyl Analogues and Other Novel Synthetic Opioids (London: NEPTUNE, 2018), https://www.drugsandalcohol.ie/28675/1/NEPTUNE_The‐misuse‐of‐synthetic‐opioids.pdf.

[17] M. Bäckberg , O. Beck , K. H. Jönsson , and A. Helander , “Opioid Intoxications Involving Butyrfentanyl, 4‐fluorobutyrfentanyl, and Fentanyl From the Swedish STRIDA Project,” Clinical Toxicology 53 (2015): 609–617. 26083809 10.3109/15563650.2015.1054505

[18] A. D. Le and S. K. Alzghari , “Systematic Review of the Clinical Consequences of Butyrfentanyl and Corresponding Analogues,” Interdisciplinary Toxicology 12 (2019): 83–88. 32206028 10.2478/intox-2019-0009 PMC7071838

[19] M. Kacela , J. Wojcieszak , and J. B. Zawilska , “Use of Fentanyl, Butyrfentanyl and Furanylfentanyl as Discussed on Polish Online Forums Devoted to ‘Designer Drugs’,” Psychiatria Polska 30 (2022): 355–372. 10.12740/PP/OnlineFirst/128156 35988080

[20] Drug Enforcement Administration, Department of Justice, Schedules of Controlled Substances . Placement of butyryl fentanyl and U‐47700 into schedule I. 2018. Accessed November 25, 2024. https://www.federalregister.gov/documents/2018/04/20/2018‐08280/schedules‐of‐controlled‐substances‐placement‐of‐butyryl‐fentanyl‐and‐u‐47700‐into‐schedule‐i.

[21] C. A. Valdez , R. N. Leif , and B. P. Mayer , “An Efficient, Optimized Synthesis of Fentanyl and Related Analogs,” PLoS One 9 (2014): e108250. 25233364 10.1371/journal.pone.0108250 PMC4169472

[22] M. T. Moody , S. Diaz , P. Shah , D. Papsun , and B. K. Logan , “Analysis of Fentanyl Analogs and Novel Synthetic Opioids in Blood, Serum/Plasma, and Urine in Forensic Casework,” Drug Testing and Analysis 10 (2018): 1358–1367. 29633785 10.1002/dta.2393

[23] A. C. F. Arantes , K. F. da Cunha , and M. S. Cardoso , “Development and Validation of Quantitative Analytical Method for 50 Drugs of Antidepressants, Benzodiazepines and Opioids in Oral Fluid Samples by Liquid Chromatography‐Tandem Mass Spectrometry,” Forensic Toxicology 39 (2021): 179–197.

[24] S. Montanari , L. Davani , C. Terenzi , et al., “Fentanyl Pharmacokinetics in Blood of Cancer Patients by Gas Chromatography—Mass Spectrometry,” Journal of Pharmaceutical and Biomedical Analysis 219 (2022): 114913. 35810723 10.1016/j.jpba.2022.114913

[25] S. Strano‐Rossi , M. J. Alvarez , M. J. Tabernero , P. Cabarcos , P. Fernández , and A. M. Bermejo , “Determination of Fentanyl, Metabolite and Analogs in Urine by GC/MS,” Journal of Applied Toxicology 31 (2010): 649–654. 21132842 10.1002/jat.1613

[26] P. Armenian , K. T. Vo , J. Barr‐Walker , and K. L. Lynch , “Fentanyl, Fentanyl Analogs and Novel Synthetic Opioids: A Comprehensive Review,” Neuropharmacology 15 (2018): 121–132. 10.1016/j.neuropharm.2017.10.016 29042317

[27] M. J. Finkelstein , C. W. Chronister , C. Stanley , L. M. Ogilvie , and B. A. Goldberger , “Analysis of Acetyl Fentanyl in Postmortem Specimens by Gas Chromatography‐Mass Spectrometry (GC‐MS): Method Validation and Case Report,” Journal of Analytical Toxicology 43 (2019): 392–398. 30767008 10.1093/jat/bky108

[28] E. Sisco , A. Burns , and A. S. Moorthy , “Development and Evaluation of a Synthetic Opioid Targeted Gas Chromatography Mass Spectrometry (GC‐MS) Method,” Journal of Forensic Science 66 (2021): 2369–2380. 10.1111/1556-4029.14877 PMC9922096 34459514

[29] Q. Wei and F. H. Su , “Determination of Nine Fentanyl Drugs in Hair Samples by GC‐MS/MS and LC‐MS/MS,” ACS Omega 31 (2022): 19176–19182. 10.1021/acsomega.2c00087 PMC9202058 35721898

[30] N. S. Mahlke , V. Ziesenitz , G. Mikus , and G. Skopp , “Quantitative Low‐volume Assay for Simultaneous Determination of Fentanyl, Norfentanyl, and Minor Metabolites in Human Plasma and Urine by Liquid Chromatography‐tandem Mass Spectrometry (LC‐MS/MS),” International Journal of Legal Medicine 128 (2014): 771–778. 24997532 10.1007/s00414-014-1040-y

[31] M. F. Fogarty , D. M. Papsun , and B. K. Logan , “Analysis of Fentanyl and 18 Novel Fentanyl Analogs and Metabolites by LC‐MS‐MS, and Report of Fatalities Associated With Methoxyacetylfentanyl and Cyclopropylfentanyl,” Journal of Analytical Toxicology 42 (2018): 592–604. 29750250 10.1093/jat/bky035

[32] C. A. Valdez , “Gas Chromatography‐Mass Spectrometry Analysis of Synthetic Opioids Belonging to the Fentanyl Class: A Review,” Critical Reviews in Analytical Chemistry 52 (2022): 1938–1968. 34053394 10.1080/10408347.2021.1927668

[33] A. M. Ares‐Fuentes , R. A. Lorenzo , P. Fernández , et al., “Determination of Synthetic Opioids in Oral Fluid Samples Using Fabric Phase Sorptive Extraction and Gas Chromatography‐mass Spectrometry,” Journal of Chromatography A 25 (2022): 462768. 10.1016/j.chroma.2021.462768 34974368

[34] N. Gilbert , L. H. Antonides , C. J. Schofield , et al., “Hitting the Jackpot‐ development of Gas Chromatography‐mass Spectrometry (GC‐MS) and Other Rapid Screening Methods for the Analysis of 18 Fentanyl‐derived Synthetic Opioids,” Drug Testing and Analysis 12 (2020): 798–811. 31989755 10.1002/dta.2771

[35] N. Misailidi , S. Athanaselis , P. Nikolaou , et al., “A GC‐MS Method for the Determination of Furanylfentanyl and Ocfentanil in Whole Blood With Full Validation,” Forensic Toxicology 37 (2019): 238–244. 30636990 10.1007/s11419-018-0449-2 PMC6314982

[36] K. B. Palmquist and M. J. Swortwood , “Quantification of Fentanyl Analogs in Oral Fluid Using LC‐QTOF‐MS,” Journal of Forensic Science 66 (2021): 1871–1878. 10.1111/1556-4029.14813 34287912

[37] E. G. de Campos , B. R. B. da Costa , F. S. Dos Santos , et al., “Alternative Matrices in Forensic Toxicology: A Critical Review,” Forensic Toxicology 40 (2022): 1–18. 36454488 10.1007/s11419-021-00596-5 PMC9715501

[38] V. Vindenes , B. Yttredal , E. L. Oiestad , et al., “Oral Fluid Is a Viable Alternative for Monitoring Drug Abuse: Detection of Drugs in Oral Fluid by Liquid Chromatography‐tandem Mass Spectrometry and Comparison to the Results From Urine Samples From Patients Treated With Methadone or Buprenorphine,” Journal of Analytical Toxicology 35 (2011): 32–39. 21219701 10.1093/anatox/35.1.32

[39] J. P. Pascali , S. Dagoli , M. Antonioni , et al., “Oral Fluid Analysis to Monitor Recent Exposure to Synthetic Cannabinoids in a High‐risk Subpopulation,” Journal of Forensic Science 67 (2022): 1932–1937. 10.1111/1556-4029.15067 35642776

[40] L. Anzillotti , F. Marezza , L. Calò , et al., “Determination of Synthetic and Natural Cannabinoids in Oral Fluid by Solid‐phase Microextraction Coupled to Gas Chromatography/Mass Spectrometry: A Pilot Study,” Talanta 201 (2019): 335–341. 31122432 10.1016/j.talanta.2019.04.029

[41] F. Bianchi , S. Agazzi , N. Riboni , et al., “Novel Sample‐substrates for the Determination of New Psychoactive Substances in Oral Fluid by Desorption Electrospray Ionization‐High Resolution Mass Spectrometry,” Talanta 202 (2019): 136–144. 31171161 10.1016/j.talanta.2019.04.057

[42] B. Sankowski , S. Michorowska , E. Raćkowska , M. Sikora , and J. Giebułtowicz , “Saliva as Blood Alternative in Therapeutic Monitoring of Teriflunomide‐Development and Validation of the Novel Analytical Method,” International Journal of Molecular Sciences 23 (2022): 9544. 36076939 10.3390/ijms23179544 PMC9455247

[43] S. Casati , M. Binda , P. Dongiovanni , et al., “Recent Advances of Drugs Monitoring in Oral Fluid and Comparison With Blood,” Clinical Chemistry and Laboratory Medicine 61 (2023): 1978–1993. 37302088 10.1515/cclm-2023-0343

[44] A. Shafi , A. J. Berry , H. Sumnall , D. M. Wood , and D. K. Tracy , “New Psychoactive Substances: A Review and Updates,” Therapeutic Advances in Psychopharmacology 10 (2020): 1–21. 10.1177/2045125320967197 PMC7750892 33414905

[45] M. Andresen Bergström , H. Lövgren , A. Abrahamsson , et al., “Rethinking Drug Analysis in Healthcare: High‐Throughput Analysis of 71 Drugs of Abuse in Oral Fluid Using Ion Mobility—High Resolution Mass Spectrometry,” Journal of Analytical Toxicology 46 (2022): 765–775. 34746960 10.1093/jat/bkab114

[46] X. Wang , Z. Hua , Z. Yang , et al., “Low‐temperature Plasma‐probe Mass Spectrometry‐based Method for Determination of New Psychoactive Substances in Oral Fluid,” Rapid Communications in Mass Spectrometry 32 (2018): 913–918. 29536614 10.1002/rcm.8112

[47] H. Elmongy and M. Abdel‐Rehim , “Saliva as an Alternative Specimen to Plasma for Drug Bioanalysis: A Review,” Trends in Analytical Chemistry 83 (2016): 70–79.

[48] European Medicines Agency . ICH guideline M10 on bioanalytical method validation and study samples.

